# Prevalence and determinants of excessive daytime sleepiness among men who have sex with men in Nepal: A cross-sectional study

**DOI:** 10.1097/MD.0000000000042341

**Published:** 2025-08-01

**Authors:** Kiran Paudel, Kamal Gautam, Manasi Sharma, Jeffrey A. Wickersham, Md. Safaet Hossain Sujan, Toan Ha, Michael M. Copenhaver, Tara Ballav Adhikari, Roman Shrestha

**Affiliations:** a Nepal Health Frontiers, Kathmandu, Nepal; b Department of Allied Health Sciences, University of Connecticut, Storrs, CT; c Manmohan Memorial Institute of Health Science, Tribhuvan University, Kathmandu, Nepal; d Department of Internal Medicine, Section of Infectious Diseases, Yale School of Medicine, New Haven, CT; e Department of Infectious Diseases and Microbiology, School of Public Health, University of Pittsburgh, Pittsburgh, PA; f Department of Public Health, Aarhus University, Aarhus C, Denmark.

**Keywords:** depressive symptoms, excessive daytime sleepiness, hypersomnia, men who have sex with men, Nepal, sleep disorder

## Abstract

Excessive daytime sleepiness (EDS) is a notable public health concern that needs comprehensive screening and effective care to mitigate potential adverse consequences. However, our understanding about EDS among men who have sex with men (MSM), particularly in low- and middle-income countries, remains limited or inadequate. Therefore, this study aims to assess the prevalence of EDS and identify the factors associated with EDS among MSM in Nepal. In Kathmandu, Nepal, a respondent-driven cross-sectional survey was carried out among 250 MSM, aged ≥ 18 years old. EDS, depressive symptoms, and social support were measured using the Epworth Sleepiness Scale (ESS-8), Patient Health Questionnaire (PHQ-9), and Oslo Social Support (OSS-3) scale, respectively. Multivariable logistic regression analysis was performed to identify potential factors associated with the outcome variable. The adjusted odds ratio (aOR) was calculated with a 95% confidence interval (CI). The prevalence of EDS among the study participants was 11.9%. MSM with moderate to severe depressive symptoms (aOR: 6.9; 95% CI: 2.5–18.7), strong social support (aOR: 4.9; 95% CI: 1.1–21.8) and disclosed sexual orientation to health care providers (aOR: 3.9; 95% CI: 1.4–11.5) had higher odds of having EDS. This study highlights preliminary evidence of a moderate prevalence of EDS among Nepalese MSM. Notably, depressive symptoms have been identified as a significant risk factor for higher scores on the ESS. This study should serve as a groundwork for future research on sleep-related disorders among MSM in Nepal.

## 1. Introduction

Excessive daytime sleepiness (EDS), or hypersomnia, is defined as the persistent desire to sleep during the day and the inability to stay attentive in situations requiring wakefulness.^[1–4]^ Its prevalence ranges from around 2% to 40% in the adult population.^[5]^ EDS often presents itself as a risk factor for many adverse health outcomes, including depressive symptoms, anxiety, cardiovascular disease, obesity, and diabetes.^[6–9]^ In addition, it might be associated with other sleep conditions like circadian rhythm disorders, sleep deprivation, obstructive sleep apnea, narcolepsy, idiopathic hypersomnia, and insomnia.^[10,11]^

Prior research has demonstrated an association between gender, sexual orientation, and sleep difficulties, revealing that individuals from sexual and gender minority groups (e.g., men who have sex with men; MSM) often report more problems with sleep quality and more frequent use of sleep medication than cisgender heterosexual participants.^[12–14]^ Sleep health research among sexual and gender minorities like MSM is gaining prominence, shedding light on the unique challenges faced by these individuals.^[14]^ Multilevel factors that are distinctly unique to MSM, such as the experience of persistent stigma, discrimination, social exclusion, violence, and ostracism within their families and society^[15,16]^ place them at increased risk of EDS. Previous studies have shown that high levels of depressive symptoms, suicide ideation, hazardous drinking, and illicit drug use may contribute to poor sleep health.^[17–19]^ Additionally, compared to their heterosexual counterparts, Nepali MSM were more likely to report depressive symptoms, suicide ideation, and parental neglect.^[16,20–22]^ Collectively, existing literature indicates that sleep health could influence the susceptibility to HIV infection among MSM by affecting their substance use patterns, sexual behaviors, and mental health.

Limited information exists regarding EDS among MSM and its determinants. To the best of our knowledge, no study in Nepal has explored daytime sleepiness among MSM, and there is a lack of evidence investigating the relationship between demographics, sexual behavior, substance abuse, mental health, and EDS. Existing studies from Nigeria and the USA have explored this aspect in MSM,^[18,23]^ but the Nepali context presents unique and distinct factors that may influence the outcomes differently. Due to Nepal’s unique socio-cultural context, existing studies from other countries may not be directly applicable. Similarly, the consequences of EDS extend beyond reduction in efficiency to difficulties in concentration and memory retention.^[24]^ Thus, this study aims to assess the prevalence of EDS and identify the factors associated with EDS among MSM in Nepal, providing insights that can inform interventions to enhance sleep quality, daytime functioning, and overall well-being among MSM in Nepal and other LMICs with similar social and cultural settings.

## 2. Methods

### 2.1. Study design and setting

Our study, Aayan (translates as “gift of the god”), was a cross-sectional study conducted in collaboration with BDS among MSM residing in the Kathmandu Valley of Nepal who were at least 18 years old and understood Nepali or English.^[25–27]^ The Kathmandu Valley comprises 3 districts: Kathmandu, Bhaktapur, and Lalitpur. Kathmandu, the densely populated and largest metropolitan city, is also the national capital, whereas Bhaktapur and Lalitpur are the neighboring districts in the valley.

### 2.2. Participants and procedure

The present study is part of a larger population-based HIV/AIDs bio-behavioral survey.^[15,27,28]^ Previous research among MSM in Nepal recruited samples between 100 and 350 MSM.^[16,21,29]^ We mirror these studies with a representative sample of MSM in Kathmandu Valley in Nepal, within the constraints of recruitment challenges for this hard-reach population.

This study employed RespondentDriven Sampling (RDS), a proven method to access and recruit hidden and hard-to-reach populations.^[30]^ Between October and December 2022, 250 MSM were enrolled, and recruitment chains began with 5 purposively selected MSM “seeds.” These seeds were chosen based on recommendations from community-based organizations working with MSM, ensuring socio-demographic and geographic representation. Upon completing the interviewer-administered questionnaire, each seed received 5 recruitment coupons to recruit potential participants. Subsequent participants were given 5 coupons each to continue the recruitment.

Trained research assistants conducted face-to-face interviews with the participants using Qualtrics^xm^ in a private room of the BDS office to ensure confidentiality. Each session lasted approximately 40 minutes, allowing for a comprehensive exploration of the research topics. Each participant received an incentive of 1000 Nepalese Rupee (~ USD 8) for study participation. Additionally, for every 5 eligible peers successfully enrolled in the study, participants received an extra 500 Nepalese Rupee (~ USD 4).

The study protocol received ethical approval from the ethical review board of Nepal Health Research Council, Kathmandu, Nepal *(Registration Number: 239/2022 P*) and the institutional review board at the University of Connecticut. All procedures involving human participants adhered to ethical standards and the Helsinki Declaration of 1964, covering the study’s goal, protocols, objectives, confidentiality and anonymity of data, and participants’ right to withdraw at any time.

## 3. Measures

### 3.1. Participant characteristics

Participant characteristics included age, sexual orientation, religion, level of education, employment status, monthly income, relationship status, family pressure to marry female, disclosure of sexual orientation at the workplace, disclosure of sexual orientation to the healthcare provider, disclosure of sexual orientation to family members, alcohol use in the past 12 months, smoking status, sexual activity in the past 6 months and condom use in the past 6 months. The dependent and independent variables are presented in Table 1.

**Table 1:** Study variables.

Variables	Categories of variable
Socio-demographic characteristics	
Age	<25 years, ≥25 years
Province of birth	Bagmati, outside Bagmati
Religion	Hindu, Buddhist, and others. Adopted from Nepal’s Health Management Information System
Level of education	Less than high school, high school, and above
Monthly income	<NRs . 20,000 (~USD 150), ≥20,000 and above
Employment	Yes, no
Sexual orientation	Gay, Bisexual
Relationship status	Single, With Partner
Behavioral characteristics	
Substance use within 6 mo	Yes, no
Ever smoked	Yes, no
Alcohol use within 12 mo	Yes, no
Ever engaged in sex work	Yes, no
Disclosed sexual orientation at the workplace	Yes, no
Disclosed sexual orientation to family members	Yes, no
Disclosed sexual orientation to healthcare providers	Yes, no
Health-related and psychosocial characteristics	
Depressive symptoms	None to mild (0–9), Moderate to Severe (10–27) based on the Patient Health Questionnaire (PHQ-9 scale)
Daytime sleepiness	Normal (0–10), Excessive daytime sleepiness symptoms (11–24) based on the Epworth Sleepiness Scale
Social support	Poor (3–8), Moderate (9-11), and Strong (12–14) social support based on Oslo Social Support, OSS-3^[31]^
Family pressure to marry a female	Yes, no
Ever tested HIV	Yes, no
Health coverage	Yes, no

OSS *=* Oslo Social Support, NRs = Nepalese Rupee, PHQ *=* Patient Health Questionnaire, USD *=* United States dollar.

### 3.2. Excessive daytime sleepiness

An 8-item Epworth Sleepiness Scale (ESS) assessed self-reported daytime sleepiness. On a 4-point scale (0-3), where “0” denotes no sleepiness, whereas “3” denotes substantial sleepiness], participants were asked to rate their chances of drifting off to sleep while engaging in 8 activities. The ESS score (range: 0 to 24) was divided into 2 categories: Normal (ESS score 0–10) and EDS symptoms (ESS score 11–24).^[32,33]^

### 3.3. Patient Health Questionnaire (PHQ-9)

The Patient Health Questionnaire (PHQ-9) scale, which corresponds to the DSM-IV Diagnostic Criteria, was used for the assessment of individuals’ depressive symptoms, fluctuating levels of hunger, trouble focusing, and suicidal ideation in the last 2 weeks. In the PHQ-9 instrument, each of the 9 DSM-IV criteria received a score from “0” (not at all) to “3” (nearly every day). A composite score of 0 to 27 was calculated, and a score of > 9 was regarded as having moderate to severe depressive symptoms.^[34]^

### 3.4. Oslo social support

The Oslo Social Support Scale (OSS-3) assessed participants’ social support levels. The 3-item scale had sum scores ranging from 3 to 14, and those score was categorized as poor (3–8), moderate (9–11), and strong (12–14) in terms of the social support.^[31]^

### 3.5. Statistical analysis

Data was analyzed using STATA version 17.0 (StataCorp LLC, College Station, Texas.). Descriptive statistics summarized the sample characteristics, while the Chi-square test evaluated the relationship between categorical independent variables and dependent variables. Multivariable logistic regression analysis identified potential factors associated with the outcome variable. The adjusted odds ratio (aOR) was calculated with a 95% confidence interval (CI), and a *P*-value < 0.05 was considered statistically significant. Bivariate analysis determined variables for inclusion in the adjusted regression analysis, with a 10% significance level as the criterion.^[35]^

In the bivariate analysis of EDS with all independent variables, the following variables educational status, relationship status, social support, family pressure to marry female, depressive symptoms, disclosed sexual orientation at the workplace, disclosed sexual orientation to family members, disclosed sexual orientation to health care providers, alcohol consumption in last 12 months, ever smoked, and condomless sex in past 6 months showed *P* value was <0.1 Then these variables were included for the multivariable logistic regression. The variance inflation factor was calculated before fitting into the model for each psychometric scale, which showed no evidence of multicollinearity.

## 4. Results

### 4.1. Characteristics of the study participants

Participant characteristics are described in Table 2. The mean age of participants was 27.6 [SD ± 8.9] years, with a majority being single (64.4%), unemployed (56.6%), and had completed a high school degree and above (58.0%). The majority of participants reported having used substances within the past 6 months (84.8%), ever smoked tobacco (72.8%), and used alcohol in the past 12 months (71.6%). Almost half of the participants reported engaging in condomless sex in the past 6 months (48.4%). Of the total participants, 19.6% reported moderate to severe depressive symptoms, and 49.6% reported poor social support.

**Table 2:** General characteristics of the participants.

Variables	Number	Percentage
Age		
Mean ± SD	27.6 ± 8.9	
<25 yr	127	50.8
≥25 yr	123	49.2
Province of birth		
Bagmati	148	59.2
Outside	102	40.8
Religion		
Hindu	173	69.2
Buddhist	54	21.6
Others	23	9.2
Level of education		
Less than high school	105	42.0
High school and above	145	58.0
Monthly income		
Less than NRs 20,000 (~USD 150)	113	45.2
NRs 20,000 and above	137	54.8
Employment		
Yes	106	42.4
No	144	56.6
Sexual orientation		
Gay	158	63.2
Bisexual	92	36.8
Relationship status		
Single	161	64.4
With partner	89	35.6
Substance use within the last 6 mo		
Yes	212	84.8
No	38	15.2
Ever smoked		
Yes	182	72.8
No	68	27.2
Alcohol use within the last 12 mo		
Yes	179	71.6
No	71	28.4
Never had condomless sex in the last 6 mo (n=182; sex in the last 6 mo)		
Yes	94	51.6
No	88	48.4
Ever engaged in sex work		
Yes	55	22.0
No	195	78.0
Moderate to severe depressive symptoms		
Yes	49	19.6
No	201	80.4
EDS		
Yes	29	11.6
No	221	88.4
Social support		
Poor	124	49.6
Moderate	108	43.2
Strong	18	7.2
Ever tested HIV		
Yes	33	13.2
No	217	86.8
Health coverage		
Yes	199	79.6
No	51	20.4
Family pressure to marry a female		
Yes	123	49.2
No	127	50.8
Disclosed sexual orientation at the workplace		
Yes	55	22.0
No	195	78.0
Disclosed sexual orientation to family members		
Yes	86	34.4
No	264	65.6
Disclosed sexual orientation to the health care providers		
Yes	62	24.8
No	188	75.2

EDS *=* excessive daytime sleepiness, HIV = human immunodeficiency virus, SD *=* standard deviation, USD *=* United States dollar

### 4.2. Factors associated with EDS

In the multivariable analysis, the factors associated with EDS were: moderate to severe depressive symptoms (aOR: 6.9; 95% CI: 2.5 to 18.7), strong social support (aOR: 4.9; 95% CI: 1.1 to 21.8), disclosed sexual orientation to health care providers (aOR: 3.9; 95% CI: 1.4 to 11.5), and family pressure to marry female (aOR: 0.3; 95% CI: 0.1 to 0.8) as shown in Table 3.

**Table 3:** Multivariable analysis of factors associated with EDS.

Variables	EDS	Adjusted odds ratio (95% CI)	*P* value
Age			
<25 yr	20 (68.9)	0.5 (0.2–1.6)	.2
25 yr and above	9 (31.1)	Ref	
Level of education			
Up to grade ten	7 (24.1)	1.2 (0.4–3.5)	
Higher secondary and above	22 (75.9)	Ref	.7
Relationship status			
Single	23 (79.3)	0.7 (0.2–2.0)	.5
With Partner	6 (20.7)	Ref	
Social support			
Poor	9 (31.0)	Ref	
Moderate	15 (51.7)	1.5 (0.5–4.2)	.4
Strong	5 (17.2)	4.9 (1.1–21.8)	**.03** *
Family pressure to marry a female			
Yes	21 (72.4)	0.3 (0.1–0.8)	**.02** *
No	8 (27.6)	Ref	
Moderate to severe depressive symptoms			
Yes	14 (48.3)	6.9 (2.5–18.7)	**<.001** **
No	15 (51.7)	Ref	
Alcohol consumption in the last 12 mo			
Yes	26 (89.6)	2.7 (0.6–10.7)	.1
No	3 (10.4)	Ref	
Ever smoked			
Yes	26 (89.6)	2.9 (0.7–12.4)	.1
No	3 (10.4)	Ref	
Condomless sex in the past 6 mo			
Yes	14 (48.3)	1.6 (0.5–4.8)	.3
No	15 (51.7)	Ref	
Disclosed sexual orientation at the workplace			
Yes	14 (48.3)	0.9 (0.3–2.8)	.9
No	15 (51.7)	Ref	
Disclosed sexual orientation to healthcare providers			
Yes	14 (48.3)	3.9 (1.4–11.5)	**.01** *
No	15 (51.7)	Ref	

Only the variables whose P values were <0.1 were included in the multivariable analysis, i.e. in this table.

EDS = excessive daytime sleepiness.

* P < .05.

** P < .001.

## 5. Discussion

This study, the first of its kind among MSM in Nepal, revealed that approximately 1 in 10 MSM experience EDS, a prevalence higher than observed in studies from Japan and Switzerland.^[5,36]^ Our findings align with studies in Korea and Australia among the general adult population,^[37,38]^ but the prevalence of EDS in this study is lower compared to studies conducted in the USA and China.^[39,40]^ This variation may be attributed to differences in settings and population characteristics. Studies conducted in Japan included the general population, whereas MSM, being a more marginalized community, showed a higher prevalence of EDS in our study. The higher prevalence of EDS in China and the USA is likely explained by factors such as the significant increase in cardiovascular disease, obesity, and overweight, as well as the extensive use of electronic devices like mobile phones, computers, and televisions, which may contribute to disturbed sleep patterns.^[39–41]^ Moreover, the cultural practice of afternoon napping in China might have influenced respondents to report higher scores on the Epworth Sleepiness Scale (ESS).

Furthermore, consistent with findings among the general population, moderate to severe depressive symptoms were found to be a significant correlate of EDS.^[42–45]^ Depressive symptoms and EDS frequently co-exist, and multiple interrelated mechanisms may explain this association. Neurobiological factors, such as dysregulation of the serotonergic system and hypothalamic pituitary adrenal axis dysfunction, contribute to disrupted sleep-wake regulation.^[46]^ Additionally, individuals with depressive symptoms often exhibit negative sleep-related cognitions and attitudes, such as preoccupation with sleep, fear of insomnia, and heightened vigilance about the consequences of poor sleep.^[45,47,48]^ Atypical depression, characterized by greater prevalence in younger individuals, may explain this association observed in our study, where the mean age was 27.6 years.^[49]^ These maladaptive beliefs can contribute to increased daytime sleepiness. The likelihood of MSM experiencing depressive symptoms and other mental health problems was higher than in the general population due to MSM-related stigma.^[49]^ Mental health interventions tailored for Nepali MSM should explore the intricate interplay between mental health and EDS, emphasizing how addressing mental health can help improve EDS and vice versa. Hence, it is imperative to implement targeted mental health interventions for MSM to lower the EDS.

This study presents novel findings indicating a positive association between disclosing sexual orientation to healthcare providers and an increased likelihood of experiencing EDS among MSM. While beyond the immediate scope of this study, the association may be explained by the inclination of MSM with co-occurring health conditions such as hypertension, diabetes, depression, and sexually transmitted infections to seek healthcare services. This prompts the disclosure of their sexual orientation for timely diagnosis and treatment. Prior research consistently reported elevated rates of EDS among individuals with comorbidities, including cardiovascular disease, diabetes, hypertension, obesity, and depression.^[39,43,50]^ To mitigate the risk of EDS, stakeholders must focus on reducing the prevalence of these co-occurring conditions like diabetes, hypertension, and obesity among MSM. Moreover, healthcare providers may have inadvertently overlooked EDS while concentrating on diagnosing and managing other symptomatic diseases. Therefore, it is crucial to pay special attention to identifying and addressing EDS in MSM who present these comorbidities during healthcare encounters.

Similarly, MSM with strong social support had higher odds of experiencing daytime sleepiness compared to those with limited social support. Our study finding contrasts with a prior study that reported no significant main effects of social support on daytime sleepiness.^[51]^ MSM receiving strong social support may be more likely to participate in social gatherings and events like late-night parties, potentially leading to insomnia and daytime sleepiness. In contrast, MSM with moderate social support had no significant differences in daytime sleepiness, possibly owing to their habit of staying awake at night with friends. Further research is necessary to elucidate the relationship between EDS and social support among MSM.

The study’s robust methodology is a notable strength, particularly its comprehensive approach to examining this minority population. Each participant was interviewed by a trained research assistant, ensuring meticulous data collection. In the analysis, we performed multivariable logistic regression, an instrumental method for mitigating the influence of confounding variables, thus enabling the identification of factors independently associated with EDS. Despite these strengths, it is important to acknowledge certain limitations of the study. Firstly, there is a lack of validation of the ESS in the Nepali version among MSM. Secondly, recall bias may be due to the study’s cross-sectional nature. Thirdly, ESS does not usually enable accurate predictions of a person’s level of drowsiness, and hence their crash risk, when driving a vehicle at some time. Therefore, affirmative evidence of EDS or an increased risk of drowsy road crashes should be sought from other sources. Lastly, the study was conducted after the COVID-19 pandemic, and its potential impact on participants should be considered. Factors such as pandemic-related stressors and changes in daily routines could have contributed to variations in sleep patterns among MSM during the study period.

## 6. Conclusion

A moderate prevalence of excessive sleepiness is observed among MSM compared to the general population with depressive symptoms identified as a significant risk factor for higher scores on the ESS. Given the significance of excessive sleepiness within this population, health-related organizations, MSM-focused organizations, and networks as well as policymakers should advocate and promote awareness programs on EDS and encourage healthcare providers to address EDS in MSM individuals presenting with depressive symptoms. This study lays the groundwork for future research on sleep-related disorders among MSM in Nepal.

## Acknowledgments

We acknowledge all the study participants for their valuable time and support in completing the study. We appreciate BDS for providing administrative support for conducting this study. We also acknowledge the financial support in part by a career development award from the National Institute on Drug Abuse (K01 DA051346) and InCHIP, University of Connecticut, for the pilot award.

## Author contributions

**Conceptualization:** Kiran Paudel, Roman Shrestha.

**Data curation:** Kiran Paudel, Kamal Gautam, Roman Shrestha.

**Formal analysis:** Kiran Paudel, Roman Shrestha.

**Funding acquisition:** Roman Shrestha.

**Investigation:** Kiran Paudel, Kamal Gautam, Roman Shrestha.

**Methodology:** Kiran Paudel, Kamal Gautam, Manasi Sharma, Jeffrey A Wickersham, Md. Safaet Hossain Sujan, Roman Shrestha.

**Project administration:** Kiran Paudel, Kamal Gautam, Jeffrey A Wickersham, Roman Shrestha.

**Resources:** Kiran Paudel, Kamal Gautam, Toan Ha, Roman Shrestha.

**Software:** Kiran Paudel, Roman Shrestha.

**Supervision:** Jeffrey A Wickersham, Toan Ha, Michael M. Copenhaver, Tara Ballav Adhikari, Roman Shrestha.

**Validation:** Roman Shrestha.

**Visualization:** Michael M. Copenhaver, Tara Ballav Adhikari, Roman Shrestha.

**Writing – original draft:** Kiran Paudel, Kamal Gautam, Manasi Sharma, Jeffrey A Wickersham, Md. Safaet Hossain Sujan, Roman Shrestha.

**Writing – review & editing:** Kiran Paudel, Kamal Gautam, Jeffrey A Wickersham, Toan Ha, Michael M. Copenhaver, Tara Ballav Adhikari, Roman Shrestha.
